# Characterization of homologous and heterologous adaptive immune responses in porcine reproductive and respiratory syndrome virus infection

**DOI:** 10.1186/1297-9716-43-30

**Published:** 2012-04-19

**Authors:** Ivan Díaz, Mariona Gimeno, Laila Darwich, Nuria Navarro, Liudmila Kuzemtseva, Sergio López, Ivan Galindo, Joaquim Segalés, Margarita Martín, Joan Pujols, Enric Mateu

**Affiliations:** 1 Centre de Recerca en Sanitat Animal (CReSA), UAB-IRTA, Campus de la Universitat Autònoma de Barcelona, 08193, Bellaterra, Barcelona, Spain; 2 Departament de Sanitat i Anatomia Animals, Universitat Autònoma de Barcelona, 08193, Bellaterra, Barcelona, Spain; 3 Institut de Recerca i Tecnologia Agroalimentàries (IRTA), Barcelona, Spain

## Abstract

The present study characterized the homologous and heterologous immune response in type-I porcine reproductive and respiratory syndrome virus (PRRSV) infection. Two experiments were conducted: in experiment 1, eight pigs were inoculated with PRRSV strain 3262 and 84 days post-inoculation (dpi) they were challenged with either strain 3262 or strain 3267 and followed for the next 14 days (98 dpi). In experiment 2, eight pigs were inoculated with strain 3267 and challenged at 84 dpi as above. Clinical course, viremia, humoral response (neutralizing and non-neutralizing antibodies, NA) and virus-specific IFN-γ responses (ELISPOT) were evaluated all throughout the study. Serum levels of IL-1, IL-6, IL-8, TNF-α and TGF-β were determined (ELISA) after the second challenge. In experiment 1 primo-inoculation with strain 3262 induced viremia of ≤ 28 days, low titres of homologous NA but strong IFN-γ responses. In contrast, strain 3267 induced longer viremias (up to 56 days), higher NA titres (≤ 6 log_2_) and lower IFN-γ responses. Inoculation with 3267 produced higher serum IL-8 levels. After the re-challenge at 84 dpi, pigs in experiment 1 developed mostly a one week viremia regardless of the strain used. In experiment 2, neither the homologous nor the heterologous challenge resulted in detectable viremia although PRRSV was present in tonsils of some animals. Homologous re-inoculation with 3267 produced elevated TGF-β levels in serum for 7–14 days but this did not occur with the heterologous re-inoculation. In conclusion, inoculation with different PRRSV strains result in different virological and immunological outcomes and in different degrees of homologous and heterologous protection.

## Introduction

One of the main obstacles for the development of new vaccines of greater efficacy against porcine reproductive and respiratory syndrome virus (PRRSV) is the limited understanding of the mechanisms involved in protection [1-4]. Up to now, most studies have focused in the development of neutralizing antibodies (NA) and to virus-specific interferon-γ secreting cells (IFN-γ-SC) as the main correlates of protection [5-10] although the precise role of these elements is not well understood. Cross neutralization experiments have shown that cross reactivity between different PRRSV strains can be low and even some PRRSV strains seem not to induce a neutralizing response at all [11,12]. Moreover, little is known about cell mediated responses in heterologous challenge models [8,10]. As a result, at present it is very difficult –or impossible- to predict the panel of strains or the characteristics of PRRSV isolates against which one pig is effectively protected after immunization. As a matter of fact, the common assumption is that immunity against a homologous strain is sterilizing –or almost complete- while immunity against other strains will depend, generically, on the degree of genetic/antigenic similarity between the immunizing and the infecting strains [1,13]. However, sequencing of ORF5 of a given strain and a vaccine is scarcely predictive of protection [8,14].

It is worth to note that after a careful review of the available scientific literature, there are very few characterized models of homologous/heterologous challenge considering simultaneously potential correlates of protection (NA and IFN-γ-SC), the development of clinical signs and the virological outcome of the challenge model. In the present study, we characterized the clinical and virological course and the evolution of neutralizing antibodies and interferon responses after inoculation with two PRRSV strains previously reported to be different [15,16]. We evaluated the neutralizing and IFN-γ-SC responses against a heterologous strain after immunization and we also tested the immunological responses after the homologous and heterologous challenges of previously immunized pigs.

## Materials and Methods

### Viruses

Two genotype I PRRSV strains designated as 3262 and 3267 were used in the present study. Strain 3262 was isolated in 2005 from the lung of a pneumonic piglet of a Spanish farm and strain 3267 was isolated in 2006 from serum of a boar of a Portuguese farm. Viral stocks were produced from passage *n* = 3 in porcine alveolar macrophages (PAM). Viral titrations were performed by inoculation in PAM and confirmation by immunoperoxidase monolayer assay (IPMA) [17]. Viral stocks were produced in an amount enough to allow the use of a single batch of virus for all experiments and assays reported below. Donor animals for PAM were free of PRRSV, Aujeszky’s disease virus and other major swine diseases including all internationally notifiable diseases plus *Mycoplasma hyopneumoniae* and swine influenza virus as shown by their serological status. The animals had maternal antibodies against porcine circovirus type 2 (PCV2) but were free of virus as determined by PCR using blood samples. PAM and viral stocks used were shown to be free of PCV2, *Mycoplasma hyopneumoniae*, hepatitis E virus and Torque teno sus virus (TTSuV) by PCR as reported before [18-21]. Strains used in the present study have been characterized in vitro before with regards to their impact on the phenotype of antigen presenting cells and have been sequenced from ORF1a to ORF7 [15,16] (Genbank accession numbers: JF276431 and JF276435). Overall genetic similarity between strains was 88.5 % [15]. Briefly, strain 3262 is known to induce IL-10 and TNF-α release in dendritic cells while strain 3267 is unable to induce any of those cytokine in the same system [16]. Strain 3262 also harbours a 74 aa deletion in in nsp2 [15].

### Experimental design and animals

All experiments involving pigs were done under the approval of the Ethics Commission for Human and Animal Experimentation of the Universitat Autònoma de Barcelona (approval n° 665). Animals were kept in approved experimental facilities and were subjected to veterinary supervision for health and welfare. Handling of pigs was done by veterinarians or trained personnel allowed to do so under the Spanish and European Union regulations. If necessary, animals were sedated for decreasing handling stress. Euthanasia was performed by pentobarbital overdose according to European Union and Spanish regulations.

The study was developed in two consecutive experiments (Table 1) carried out in biosafety level 3 facilities of CReSA one immediately after the other. Experiments were carried out using pigs of the same origin (same farm, same genetic background, etc.) and were housed under the same controlled environmental conditions. Source farm was historically free of PRRSV and pigs were re-confirmed to be free of PRRSV by ELISA (Herdcheck 2XR, Idexx Laboratories) and RT-PCR [22] and free of PCV2 by PCR [19]. In both experiments, upon arrival at the experimental facilities, pigs were ear-tagged, weighed, bled and randomly distributed (random numbers) in two pens; then, piglets were left to acclimatize for one week. Inoculations were always done by the intranasal route using 2 mL (1 mL/nostril) of the appropriate PRRSV strain at a dose of 5 × 10^5.0^ TCID_50_/mL. Control animals received 2 mL of sterile RPMI by the intranasal route when necessary.

**Table 1:** Experimental design

	**Groups**	**1**^**st**^Challenge* (0 pi)	**2**^**nd**^Challenge* (84 pi)
**Experiment 1** 20 pigs	A	strain 3262	Group A + A (*n* = 4)
			Homologous challenge: strain 3262
	14 pigs^†^		Group A + B (*n* = 4)
			Heterologous challenge: strain 3267
	C1	Placebo	Group C1 + B (*n* = 2)
	6 pigs^††^		naïve controls: strain 3267
**Experiment 2**11 pigs	B	strain 3267	Group B + B (*n* = 4)
			Homologous challenge: strain 3267
	8 pigs		Group B + A (*n* = 3**)
			Heterologous challenge: strain 3262
	C2	Placebo	Group C_2_ + A (*n* = 3)
	3 pigs		naïve controls: strain 3262

* Inoculation was performed intranasally (2 mL; 1 mL/nostril) with a dosis of 5 × 10^5.0^ TCID_50_/mL of the corresponding strain

** One pig died at 17 pi in the first inoculation.

† Six pigs were euthanized; three at 15 pi and three at 35 pi.

†† Four pigs were euthanized: two at 15 pi and two at +35 pi.

Also, at the start and at the end of the experiment serum samples were examined by ELISA for antibodies against PCV2 (Ingezim PCV2, Ingenasa, Madrid, Spain) and hepatitis E virus [23]. Presence of TTSuV was assessed by PCR as reported [21]. Other examined pathogens were Aujeszky’s disease virus (Herdcheck PRV gE, Idexx Laboratories, Barcelona, Spain), *Mycoplasma hyopneumoniae* (Ideia *Mycoplasma Hyopneumoniae* EIA KIT, Oxoid, Cambridge, UK) and swine influenza virus (Civtest suis influenza, Hipra Laboratories, Amer, Spain). All animals were seronegative to those pathogens when the experiments ended.

In experiment 1, 20 three-week-old piglets were divided in two groups: A (*n* = 14) and C1 (*n* = 6). Group A was inoculated with strain 3262 and group C1 received RPMI as a placebo. At 15 dpi, three piglets of group A and two animals of group C1 were euthanized and subjected to a complete necropsy. On day 35 post-inoculation (pi), three further A piglets and two control animals (C1) were euthanized and subjected to necropsy. Remaining animals (eight in group A and two in C1) were followed up until day 84 pi. At that time, four A piglets were randomly selected (random numbers), separated in a different isolation box and inoculated with strain 3262 (group A + A corresponding to the homologous challenge (HoC) for experiment 1). The other four A pigs were inoculated with strain 3267 (group A + B; namely a heterologous challenge (HeC) for experiment 1). C1 pigs were challenged with PRRSV strain 3267 (from now on C1 + B). All animals were monitored for the following two weeks and then were euthanized and subjected to necropsy (day 98 pi, 14 days post-second challenge).

In experiment 2, 11 three-week-old animals were used. Initially two groups were formed: B (*n* = 8) and C2 (*n* = 3). B animals were inoculated with strain 3267 and animals in C2 received RPMI. In this case sequential necropsies at days 15 and 35 pi were not done. Eighty four days later (day 84 pi), four B pigs were inoculated again with strain 3267 (B + B group, HoC for experiment 2) and 3 piglets (one piglet died at day 17 pi after the initial challenge) received strain 3262 (B + A, HeC for experiment 2). C2 animals were also challenged then with PRRSV strain 3262. Animals were monitored for the following two weeks as above.

### Clinical follow-up and sample taking

In both experiments, animals were clinically examined on arrival and then were monitored for the development of clinical signs including fever: 18 days in experiment 1 or for 36 days in experiment 2. This difference in the periods for which rectal temperatures were recorded was attributable to the fact that feverish pigs were still observed at 14 pi in experiment 2. Weights were recorded weekly from day 0 to 42 pi. Blood samples were taken (siliconized and heparinized tubes) weekly.

### Pathology

At necropsy, samples were systematically taken from lungs (portions of all lobes of the right lung plus the accessory lobe), submandibular, tracheobronchial, mesenteric and inguinal superficial lymph nodes, tonsils, thymus and spleen. If relevant gross lesions were found besides those organs, additional *ad hoc* samples were taken. All organs were sampled in duplicate; one sample was fixed in 10 % neutral buffered formalin and embedded in paraffin and the other was frozen at −80°C until needed for further analysis. Tissues were examined histopathologically (haematoxylin/eosin staining) for evaluating the nature and severity of lesions. In order to avoid biases, this examination was done in a blinded fashion by three pathologists. For interstitial pneumonia, a 4 grade score was used: 0 (no lesion), 1 (mild), 2 (moderate) and 3 (severe). Similar grading was used for the evaluation of lymphoid hyperplasia (white pulp hyperplasia in spleen and follicular hyperplasia in lymph nodes and tonsil). For thymus, the ratio cortex to medulla was calculated and the presence of tingible body macrophages was evaluated in thymus (0 to 4 scale, 0 = none; 1 = low, 2 = moderate, 3 = high, 4 = very high). Also, lymphoid tissues were analysed by in situ hybridization (ISH) to assess presence of PCV2 as previously described [25]. In case that in the course of necropsies gross lesions indicative of bacterial infections were found, appropriate microbiological cultures were performed.

### Virological analysis

The evolution of viremia was initially assessed by viral isolation in PAM and by nested RT-PCR (nRT-PCR). For this, blood samples were serially diluted from 10^0^ to 10^-4^ and inoculated in PAM cultures (50 μL in quadruplicates). Isolation was verified at day 3 pi in PAM by means of IPMA as reported above. For each animal, serum samples were re-tested for the presence of PRRSV by nRT-PCR [6] starting in the last sample (in chronological order) producing a positive viral isolation. For formalin fixed tissues, PRRSV presence was determined initially by means of an immunohistochemical technique (IHC) [25]. For lungs, tonsils and spleen, samples producing negative IHC were then re-tested using the same nRT-PCR as above, adapting RNA extraction procedure to frozen tissues.

In order to examine if viremia at late times after the different challenges corresponded to the predominance of neutralization escape mutants, RNA was extracted from selected samples and analysed for ORF4 and ORF5 specific sequences according to previously reported methods [15]. In order to assess if virus present in tissues after the second challenge corresponded to persistent infection or not, sequencing for ORF5 was performed on nRT-PCR positive tonsils collected from HeC pigs.

### Humoral response

Serum samples were analysed by means of a commercial ELISA (HerdCHeck 2XR, Idexx Laboratories). Results were reported as a ratio (S/P) of optical densities between the results of a given sample and the positive control included in the kit (cut-off: S/P ≥ 0.4). All serum samples were tested the same day and in the same batch of plates.

Also, levels of NA were determined. Since the used PRRSV strains could not be adapted to grow in MARC-145 cells at low passages (*n* ≤ 4), viral neutralization tests (VNT) were done in PAMs. In order to test the cross-reactivity of antibodies against strains 3262 or 3267, VNT were performed in parallel with each viral strain. Briefly, sera were diluted (in duplicate) in a serial dilution (log_2_) from 1/2 to 1/256, inactivated at 56°C 30 min and incubated overnight 1:1 vol/vol at 4°C with one or the other of the abovementioned strains (2000 TCID_50_/mL). PAMs were cultured in 96 wells plates (50 000/well) with the virus-serum mixture. After 72 h of incubation, PAMs were fixed in absolute ethanol at −20°C until needed. The reaction was revealed by means of a peroxidase conjugate using a non-neutralizing monoclonal antibody against ORF5 (3AH9; Ingenasa, Madrid, Spain) and insoluble TMB (TMB/h; Milipore, Billerica, USA) as a chromogen. In order to have an assessment of the accuracy of the measurement, testing was done twice for days 42 pi, 84 pi and 98 pi (14 post second challenges). In addition, to test if negative neutralization results at 72 h post-inoculation of PAM could be due to selection and growth of a neutralization escape mutant variants or quasispecies present in the original virus stock, a panel of selected sera yielding negative results in the VNT were used in a second VNT that was performed as above but revealed after 12 h of incubation of PAM with the virus/serum mixture in order to avoid propagation of the hypothetical neutralization escape mutants.

### IFN-γ ELISPOT

Evaluation of the cell-mediated immune response (CMI) was done by the IFN-γ ELISPOT using peripheral blood mononuclear cells (PBMC) [6,26] at days 0, 21, 42, 63, 70, 77 and 84 pi plus on days 91 and 98 (7 and 14 post second challenges, respectively). In order to evaluate the in vitro homologous and heterologous responses in the IFN-γ ELISPOT, PBMC were stimulated in parallel (500 000 PBMC/well, 3 wells per pig and stimulus) with strains 3262 and 3267 at multiplicity of infection (m.o.i.) of 0.01. Unstimulated cells and phytohemagglutinin-stimulated controls (10 μg/mL) were also included. PRRSV-specific corrected frequencies of IFN-γ-SC were calculated by subtracting counts of spots in unstimulated wells from counts in virus-stimulated wells. Frequencies of IFN-γ-SC were expressed as responding cells in 10^6^ PBMC.

### Cytokine ELISAs

In both experiments serum samples were taken at days 0, 1, 2, 3, 7 and 14 post second challenges (first challenge for control groups C1 and C2) and tested by ELISA for determining serum levels of TNF-α, IL-1, IL-6, IL-8, IL-10 and TGF-β. ELISAs were performed using commercial pairs of monoclonal antibodies according to manufacturer’s instructions: TNF-α, IL-1, IL-6 and IL-8 (Porcine TNF-α, IL-1, IL-6 and IL-8 DuoSet, respectively; R&D Systems, Minneapolis, USA) and IL-10 and TGF-β (IL-10 Swine Antibody pair and TGF-β1 Multispecies ELISA Kit; Invitrogen). The cut-off of each ELISA was calculated to be equal to the mean optical density (OD) of negative controls plus three standard deviations (cut-off = 32 pg/mL for IL-6, IL-10, TNF-α, TGF-β1; cut-off = 62 pg/mL for IL-1 and IL-8). For a given sample, the OD was then transformed to concentration by applying a linear regression formula calculated from the results of the standards provided in each kit. In order to account for the pre-2nd challenge levels of a given cytokine, values (pg/mL) obtained with samples of day 0 (84 pi) were subtracted to the values obtained at days 1, 2, 3, 7 or 14 producing thus a corrected value (Corrected value for cytokine X = Concentration of cytokine X _day n_ - Concentration of cytokine X _day 0_).

### Statistical analysis

Statistics were performed using StatsDirect v2.7.7. Kruskal-Wallis test (Conover-Inman method for multiple comparisons) was used for comparisons of means between groups and the Log Rank & Wilcoxon test was used for comparing the duration of viremias. Comparison of the proportion of infected tissues was determined by the χ^2^ test (Fisher’s exact test). EpiCalc 2000 was used for calculation of confidence intervals of proportions.

## Results

### Clinical follow-up and weight gains

**Figure 1:**
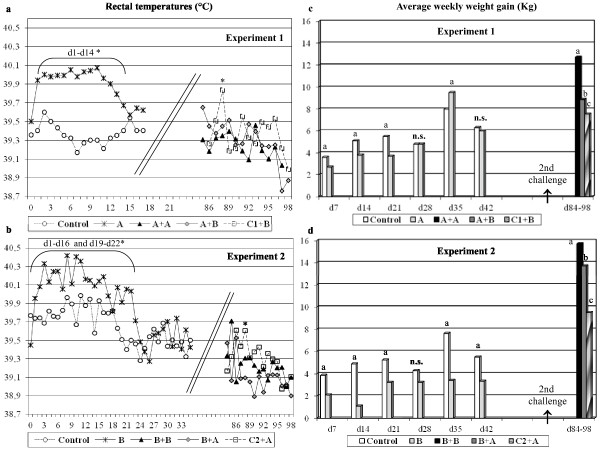
Rectal temperature and body weight gains after inoculation with PRRSV. Evolution of average rectal temperatures and body weight gains in experiments 1 (figure a-c) and 2 (b-d). In experiment 1, animals received initially (day 0 pi) PRRSV strain 3262 (A; black crosses, light grey bars) or were mock-inoculated (Control; empty circles, empty bars). At day +84 pi, four pigs were challenge with strain 3262 again (A + A; black triangles, black bars) or received a heterologous challenge with strain 3267 (A + B; grey rhombus or dark grey bars). Naïve pigs were challenged with strain 3267 (C1 + B; empty squares or diagonal line bars). In experiment 2, pigs were initially inoculated with strain 3267 (B) and later challenged with 3267 (B + B) or 3262 (B + A). Controls were included (Control and C2 + A). Significant (*p* < 0.05) differences in body temperatures are marked with an asterisk. For weight gains (right side of the figure), bars with a letter above show significant (*p* < 0.05) differences.

Figure 1 shows the evolution of body temperatures and weight gains in experiments 1 and 2. In both experiments inoculation of PRRSV induced moderate fever, up to 40.4°C, during at least the first 14 days pi. In the case of experiment 2, fever was more prolonged and body temperatures of inoculated animals were significantly higher (*p* < 0.05) compared to controls until day 22 pi.

In experiment 1, respiratory signs were absent or very mild while in experiment 2, dullness and respiratory signs were slightly more evident including occasional coughing and laboured breath. In the second experiment one animal died at 17 pi showing gross lesions of fibrinous to fibrous pleuritis, catarrhal bronchopneumonia and interstitial pneumonia, suggesting a bacterial complication that could not be confirmed in the microbiological analysis. Regarding weight gains, in Experiment 1 inoculated animals showed decreased weight gains (*p* < 0.05) compared to controls until day 21 pi and in experiment 2 differences were noticeable until 42 pi. For the first 42 days pi, weight gains of control pigs were 33.3 Kg in experiment 1 and 31.3 Kg in experiment 2, while weight gains for virus inoculated animals in the same period were 30.5 Kg in experiment 1 and 16.3 Kg in experiment 2 (*p* < 0.05).

After the second challenge, clinical signs were absent in both experiments except in control animals (C1 and C2) that were immunologically naive and showed fever occasionally. After the second challenge weight gains were significantly different among groups (*p* < 0.05) and always animals subjected to a HoC had better gains than animals subjected to HeC or than naïve pigs inoculated for the first time.

### Pathology

In experiment 1, necropsies performed at day 15 pi showed that inoculated animals developed gross interstitial pneumonia that microscopically ranged from slight (1) to severe (3). No gross lesions were seen beyond those observed in lungs. At that time (15 pi) PRRSV was detected by IHC in lungs of all inoculated animals. Uninoculated controls did not develop gross or microscopic lesions and were free of PRRSV by IHC. At day 35 pi, inoculated pigs did not show macroscopic or microscopic signs of interstitial pneumonia and were negative to PRRSV by IHC.

Regarding the development of lesions after the second challenge in experiment 1 -that took place at day 84 pi- none of the pigs showed macroscopic evidence of interstitial pneumonia, except for one in A + B group. Microscopically (Table 2), both the HoC and the HeC groups showed similar lesions of interstitial pneumonia with scores ranging from slight to severe depending on the lung lobe examined. Control animals infected with strain 3267 for the first time (C1 + B) had similar lung lesions than the HoC or the HeC group. In experiment 2, necropsies performed two weeks after the second challenge showed that only one pig (B + A group) had signs of gross interstitial pneumonia.

**Table 2:** Results of the histopathological examination of lungs

**Experiment**	**Group**	Interstitial pneumonia*
**1**	**C1 + B**	5.0 ± 2.8^a^
	**A + A**	6.5 ± 3.8^a^
	**A + B**	6.8 ± 1.3^a^
**2**	**C2 + A**	8.7 ± 2.3^b†^
	**B + B**	6.5 ± 2.6^a^
	**B + A**	5.3 ± 0.6^a^

Different superscript letters indicate significant differences (*p* < 0.05) between the groups in a given experiment according to the Kruskal-Wallis test (Conover-Inman test for multiple comparisons). No significant differences were seen for the double challenged groups between experiment 1 and 2.

* Microscopic interstitial pneumonia was evaluated in 4 portion of lung corresponding to the apical, cardiac, diaphragmatic and intermediate lobes, scored in a 4-grade scale (0 = absent to 3 = severe). The values shown correspond to the average of the summatory of scores for each pig in a given group and the standard deviation of the summatories. With this calculation, maximum possible average is 12.

† In the case of group C2 + A; *p* = 0.06.

No differences were observed between groups regarding lymphoid hyperplasia in spleen, tonsils or lymph nodes in none of the experiments. However, density of tingible body macrophages was apparently higher in the B + A group compared to the others (not shown).

### Virological analysis

Results of the evolution of viremia are shown in Tables 3 and 4. Inoculation of naive pigs with strain 3262 (experiment 1) resulted in viremia (nRT-PCR) by day 7 pi in all animals. The proportion of viremic pigs declined afterward to 11/14 by day 14 pi (78.6 %; CI_95%_: 48.8-94.3 %), 4/11 at 21 pi (36.4 %; CI_95%_: 12.4-68.4 %) and 1/11 at 28 pi (9.1 %; CI_95%_: 0.5-42.9 %). By day 35 pi all inoculated animals had become negative in blood and tissues (nRT-PCR and IHC). Viral load in serum peaked at 7 pi (average viral titre of 10^3.1^ TCID_50_/mL) and declined later on.

**Table 3:** Detection of virus in sera by isolation in porcine alveolar macrophages and by nested RT-PCR after first challenge with PRRSV

													
**Experiment**	**Assay**	**7**	**14**	**21**	**28**	**35**	**42**	**49**	**56**	**63**	**70**	**77**	**84**
**1**	**Viral isolation (titre)**	14/14	11/14^†^	4/11	1/11	0/11	0/8	0/8	0/8	0/8	0/8	0/8	0/8
		3.1 ± 0.6	2.9 ± 0.5	2.3 ± 0.2	2.3 ± 0.0	-	-	-	-	-	-	-	-
	**nRT-PCR**	14/14	11/14	4/11	1/11	0/11	0/8	0/8	0/8	0/8	0/8	0/8	0/8
**2**	**Viral isolation (titre)**	8/8	8/8	7/7^††^*	7/7*	7/7*	3/7*	3/7*	1/7*	0/7	0/7	0/7	0/7
		3.7 ± 0.3	3.4 ± 0.5	3.2 ± 0.8	2.6 ± 0.5	2.2 ± 0.2	2.0 ± 0.3	2.0 ± 0.3	1.8 ± 0.0	-	-	-	-
	**nRT-PCR**	N.D.	N.D.	N.D.	N.D.	N.D.	4/7	4/7	3/7	0/7	0/7	0/7	0/7

Results of viral titration of sera in porcine alveolar macrophages (PAM) using the immunoperoxidase monolayer assay are expressed as number of positive pigs over the number of pigs in the group and as the mean ± standard deviation (log_10_) of titres (TCID_50_/mL) of positive pigs. *p < 0.05 for the comparison of infected groups between experiment 1 (strain 3262) and 2 (strain 3267). For the nRT-PCR results only show the number of positive pigs in the second round of the PCR. Results of control (uninoculated) pigs are not shown because they were always negative from day 0 to 84 post-inoculation.

^†^ Three pigs were euthanized at day 15 pi and three pigs more were killed at day 35 pi.

^††^ One pig died at day 17 pi.

N.D. Not done

**Table 4:** Detection of virus in sera by isolation in porcine alveolar macrophages and by nested RT-PCR after the second challenge with PRRSV

				
**Experiment**	**Group**	**Assay**	**+7 (91 pi)**	**+14 (98 pi)**
**1**	**C1 + B**	**Viral isolation**	2/2	2/2
			3.2 ± 0.6	2.0 ± 0.0
		**nRT-PCR**	2/2	2/2
	**A + A**	**Viral isolation**	0/4	0/4
		**nRT-PCR**	4/4	1/4
	**A + B**	**Viral isolation**	0/4	0/4
		**nRT-PCR**	4/4	0/4
**2**	**C2 + A**	**Viral isolation**	3/3	2/3
			3.4 ± 0.9	2.2 ± 0.1
		**nRT-PCR**	3/3	3/3
	**B + B**	**Viral isolation**	0/4	0/4
		**nRT-PCR**	0/4*	0/4
	**B + A**	**Viral isolation**	0/4	0/4
		**nRT-PCR**	0/4*	0/4

*Asterisks indicate statistical differences in the proportion of pigs found to be positive by RT-PCR in comparable groups of experiments 1 and 2 (i.e. homologous A + A versus homologous B + B).

After the second challenge at day 84 pi, all animals, regardless of the previous status of immunization or the strain used for this second challenge, became infected again as shown by results of sera by nRT-PCR (Table 4); however, viral isolation from blood was only achieved for PRRSV-naïve pigs. Examination of tissues by IHC and nRT-PCR (Table 5) revealed that 14 days after the second inoculation, PRRSV could be detected in lungs of 2/4 pigs in both the HoC and HeC groups. In contrast, a trend for a significantly higher presence of virus in tonsils was observed in the HeC compared to the HoC (*p* = 0.07).

**Table 5:** Distribution of PRRSV in tissues after the homologous or heterologous challenge by nested RT-PCR or immunohistochemistry (IHC)

					
**Experiment**	**Group**	**Assay**	**Lung**	**Tonsil**	**Spleen**
**1**	**C1 + B**	*nRT-PCR*	0/2	2/2	1/2
		*IHC*	0/2	1/2	0/2
	**A + A**	*nRT-PCR*	2/4	1/4	1/4
		*IHC*	0/4	1/4	1/4
	**A + B**	*nRT-PCR*	2/4	4/4*	3/4
		*IHC*	0/4	0/4	1/4
**2**	**C2 + A**	*nRT-PCR*	1/3	1/3	0/3
		*IHC*	0/3	0/3	0/3
	**B + B**	*nRT-PCR*	0/4	2/4	0/4
		*IHC*	0/4	0/4	0/4
	**B + A**	*nRT-PCR*	0/3	1/3	0/3
		*IHC*	0/3	1/3	0/3

*p = 0.07

In experiment 2, all animals were viremic (nRT-PCR) for the first 35 days pi and by day 56 pi 3/7 pigs were still positive (42.9 %; CI_95%_: 11.8-79.8 %). Regarding viral isolation and titration, viremia peaked at day 7 pi with an average viral load in serum of 10^3.7^ TCID_50_/mL and viral load remained above 10^3.0^ TCID_50_/mL for 21 days pi. From day 14 pi onwards, viral loads were higher in animals infected with strain 3267 compared to animals infected with strain 3262 in experiment 1 (*p* < 0.05) and duration of viremia was also statistically longer (*p* < 0.05). To test if the longer viremia seen for strain 3267 was attributable to the generation and expansion of neutralization escape mutants in the course of infection, viral isolates of day 49 pi were sequenced for the ORF4 and ORF5. Results of sequencing showed that the NE in GP4 changed during the course of the infection and that GP5 gained one potential glycosylation at N37 (DSS →NSS).

After the second challenge of day 84 pi only PRRSV-naïve pigs developed a detectable viremia that lasted until the end of the experiment (Table 4). Examination of tissues indicated however that at least two animals in the HoC group and one animal in the HeC group had become infected as demonstrated by the presence of the virus in tonsils (Table 5). Sequencing demonstrated that the virus found in the HeC experiments always corresponded to the heterologous virus and was not a remnant of the initial inoculation.

### Humoral response

**Figure 2:**
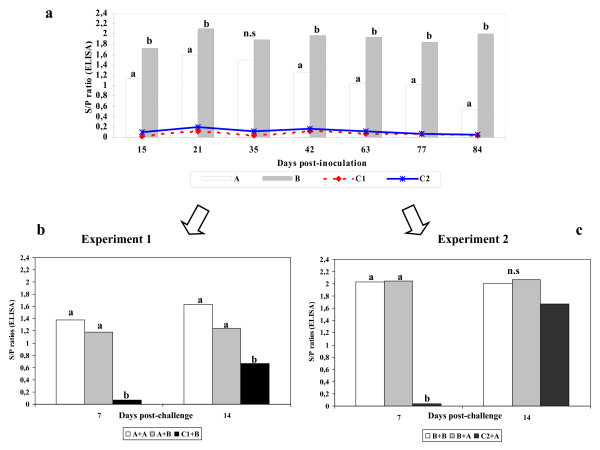
Evolution of the humoral response (ELISA) against PRRSV in experiments 1 and 2. 2a. Average sample to positive (S/P) ratios of optical densities from day +15 to +84 post-inoculation. Empty bars correspond to pigs in experiment 1 (strain 3262: A); grey bars correspond to pigs in experiment 2 (strain 3267: B). Lines show results of control (un-inoculated pigs) in both experiments. 2b. Serological evolution (S/P ratios) of pigs in experiment 1 after the second challenge (84 pi). Empty bars correspond to the homologous challenge (group A + A), grey bars to the heterologous challenge (A + B) and black bars depict results of naïve pigs inoculated for the first time with strain 3267 (C1 + B) 2c. Serological evolution (S/P ratios) of pigs in experiment 2 after the second challenge (84 pi). Empty bars: (B + B); grey bars: B + A; black bars: naïve pigs inoculated for the first time with strain 3262 (C2 + A). In all cases, different superscript letters indicated statistically significant differences (*p* < 0.05) as determined in the Kruskal-Wallis test; n.s. = non-significant.

After inoculation with either one or the other PRRSV strain, pigs rapidly developed antibodies detectable in ELISA although S/P ratios were different among groups. Thus, for the first 84 days pi (except at day 35 pi) S/P ratios were higher (*p* < 0.05) in animals inoculated with 3267 -experiment 2- than in animals inoculated with 3262- experiment 1- (Figure 2a). After the second challenge (day 84 pi), re-inoculated animals became similar in terms of S/P ratios within each one of the experiments (Figures 2b and 2c).

Development of NA against the homologous and the heterologous strains was examined in VNT using PAM (Table 6). In experiment 1, after the first inoculation none of the pigs developed NA against the inoculation strain 3262 (homologous VNT). After challenge at day 84 pi, serum of pigs re-inoculated with either 3262 or 3267 did not show neutralizing activity against strain 3262. In contrast, in experiment 2 (inoculation with strain 3267) NA against the homologous strain were demonstrated in some pigs already by day 21 pi and by day 42 all inoculated pigs were positive for NA. At day 84 pi, titres of homologous (3267) NA ranged from 3 log_2_ to 6 log_2_. After the second challenge (day 84 pi), most animals showed an increase of the homologous (3267) VNT titres by 1–2 log_2_. Naïve pigs inoculated with 3267 for the first time at 4 months of age in experiment 1 already showed NA titres in the range of 2–3 log_2_ against that strain 14 days after the inoculation (day 98).

**Table 6:** Viral neutralization test results using PRRSV strains 3262 and 3267

	**Nº of responding pigs; mean titre (log**_**2**_**) and standard deviation** **Range of titres (log**_**2**_**) for positive pigs**							
	**Strain used in the viral neutralization test**							
	**3262**				**3267**			
	**Days post-inoculation**				**Days post-inoculation**			
**Challenge group†**	**21**	**42**	**84**	**98**	**21**	**42**	**84**	**98**
**A + A**	0/4	0/4	0/4	0/4	0/4	3/4*	4/4*	4/4*
**(3262+3262)**	-	-	-	-	-	1.7 ± 0.6^a^	2.0 ± 0.8^a^	4.25 ± 1.0^b^
						(1–2)	(1–3)	(3–5)
**A + B**	0/4	0/4	0/4	0/4	0/4	2/4	2/4	4/4*
**(3262+3267)**	-	-	-	-	-	1.0 ± 0.0^a^	2.0 ± 0.0^a^	5.3 ± 0.5^b^
						(1–1)	(2–2)	(5–6)
**C1 + B**	0/2	0/2	0/2	0/2	0/2	0/2	0/2	2/2*
**(C+3267)**	-	-	-	-	-	-	-	2.5 ± 0.5
								(2–3)
**B + B**	0/4	2/4	4/4‡	4/4‡	4/4‡	4/4	4/4	4/4
**(3267+3267)**	0/4	1.0 ± 0.0	2.2 ± 1.5	2.8 ± 1.7	2.0 ± 0.8^a^	2.5 ± 1.0^a^	4.8 ± 1.0^b^	6.0 ± 0.8^b¶^
	-	(1–1)	(1–4)	(1–5)	(1–3)	(2–4)	(4–6)	(5–7)
**B + A**	0/3	0/3	3/3‡	3/3‡	1/3	3/3	3/3	3/3
**(3267+3262)**			2.0 ± 1.0	1.3 ± 0.6	1.0 ± 0.0	3.0 ± 1.0^a^	4.0 ± 1.0^a^	5.3 ± 0.6^b^
	-	-	(1–3)	(1–2)	(1–1)	(2–4)	(3–5)	(5–6)
**C2 + A**	0/3	0/3	0/3	0/3	0/3	0/3	0/3	0/3
**(C+3262)**	-	-	-	-	-	-	-	-

† Chronological sequence of strains used for inoculation is shown in parentheses.

* The asterisk show statistical differences (*p* < 0.05) for the proportion of responding pigs using one or the other PRRSV strain.

Superscript letters indicate statistical differences (*p* < 0.05; ¶ *p* = 0.08) in the average titres between different days using a given strain.

‡ The symbol indicates statistically significant differences for the proportion of responding pigs for similar groups between experiments (i.e. homologous A + A vs. homologous B + B) using the same strain.

When cross-neutralization tests were performed, the picture was different. Thus, in experiment 1 by day 84 pi sera of 6/8 animals (75 %; CI_95%_: 35.6-95.6 %) showed neutralizing activity against 3267 (heterologous in this case) but with low titres (1–3 log_2_). After the second challenge, a boost in the VNT titres against 3267 (3–6 log_2_) was seen regardless of the strain used for that second challenge. In experiment 2, sera of pigs inoculated with 3267 showed a weak neutralizing activity against 3262 (heterologous) by day 84 pi (1–4 log_2_). After the second challenge, heterologous titres against 3262 did not substantially increase except for one pig (receiving 3267 again).

### Evolution of the IFN-γ ELISPOT after the initial challenge

**Figure 3:**
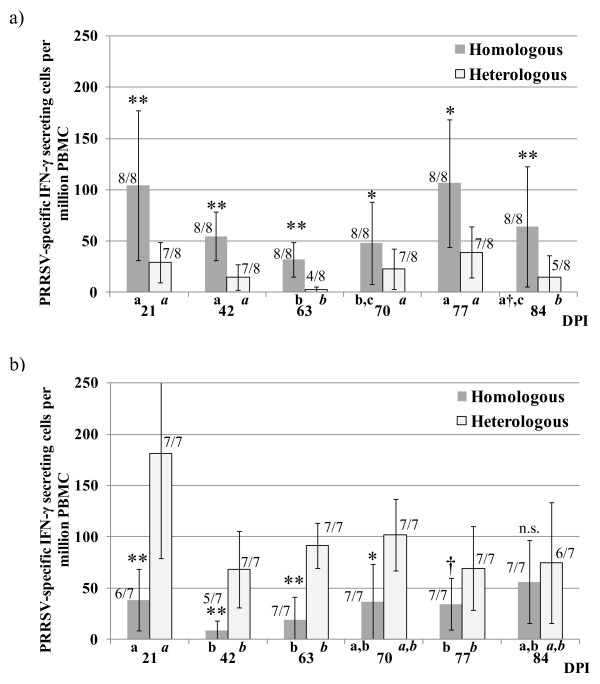
PRRSV-specific cell-mediated immune response as determined by the IFN-γ ELISPOT, days 21 to 84 pi. Peripheral blood mononuclear cells (PBMC) were stimulated with either of the PRRSV strains (3262 or 3267) used in the study. Results are shown (columns) as average frequencies of virus-specific IFN-γ secreting cells per million of PBMC obtained in the ELISPOT with the corresponding standard deviations (bars). For each experiment the results of ELISPOT are shown in two series: darker shadowed columns indicate results obtained after in vitro stimulation with the same PRRSV strain with which had been immunized initially (homologous) while lighter coloured bars show the results of in vitro stimulation with other PRRSV isolate used in the present study (heterologous). Asterisks indicate significant differences between results obtained in the homologous and the heterologous ELISPOT for each experiment (*p < 0.05; **p < 0.01; † *p* = 0.07) at a given time point. Letters indicate significant differences (*p* < 0.05) among different time points in a given series. For each time point and series, the number of responding pigs over the total number of examined pigs is shown. 3A = Experiment 1 (inoculation with strain 3262); 3B = Experiment 2 (inoculation with strain 3267). dpi = days post-inoculation.

For each of the experiments, the IFN-γ ELISPOT was performed using in parallel each of the strains included in the study in order to examine the homologous and heterologous response (Figures 3a and 3b). In experiment 1, inoculation with strain 3262, the use of the same 3262 strain in the ELISPOT revealed a peak of virus-specific IFN-γ secreting cells (SC) by day 21 pi; thereafter frequencies decreased to a low by day 63 pi. Using the strain 3267 as stimulus (heterologous stimulation in this case), the trend was similar but with significantly (*p* < 0.05) lower frequencies of responding cells in all examined days.

In experiment 2, inoculation with strain 3267, the ELISPOT using the strain 3267 produced a similar temporal trend with the lowest frequencies at days 42 and 63 pi. In this case, the use of strain 3262 (heterologous stimulation in ELISPOT), resulted in much higher frequencies (*p* < 0.05) for the first 70 days pi and almost significant differences (*p* = 0.06) were still determined by day 77 pi.

When equivalent series of IFN-γ ELISPOT results were compared between experiments; namely, homologous stimulation in experiment 1 versus homologous stimulation in experiment 2 and heterologous stimulation in experiment 1 versus heterologous stimulation in experiment 2, differences were also seen. Thus, inoculation with 3262 produced higher frequencies of IFN-γ cells (*p* < 0.05) for days 21, 42 and 77 pi compared to the inoculation with 3267 (104 ±73 vs. 38 ± 30; 54 ± 24 vs. 9 ± 9 and 106 ± 69 vs. 34 ± 25, respectively). Comparing results of heterologous stimulation in ELISPOT, the use of strain 3262 resulted in higher responses (*p* < 0.05) at days 21, 42, 63 and 70 pi (181 ± 102 vs. 29 ± 19; 68 ± 37 vs. 14 ± 12; 91 ± 22 vs. 2 ± 3 and 101 ± 34 vs. 22 ± 20).

### Evolution of the IFN-γ ELISPOT after the second challenge

**Figure 4:**
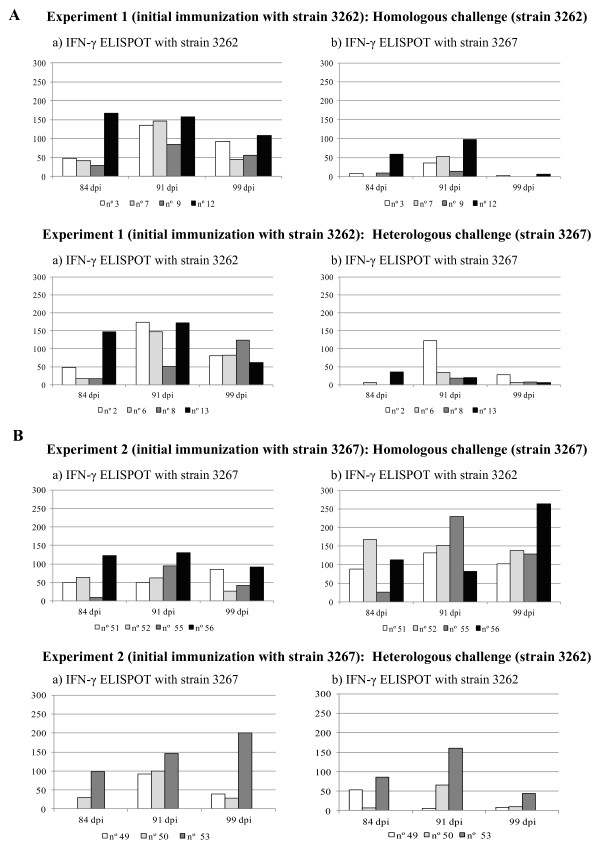
Individual responses in the IFN-γ ELISPOT after second challenge that took place at day 84 pi. Bars depict individual frequencies of virus-specific IFN-γ secreting cells after the homologous and heterologous challenge for this experiment. ELISPOTs were performed with both strains used in the present study.

Figures 4a and 4b summarize this part of the experiment. After the challenge of day 84 pi, the behaviour of the IFN-γ-SC response varied depending on the experiment. Thus, for experiment 1 (initial immunization against strain 3262), re-inoculation of PRRSV resulted in a significant boost of the IFN-γ ELISPOT responses both in the HoC and in the HeC groups by day 91 except for animals that had reached frequencies of IFN-γ SC ≥ 100 before the challenge. In this case again, use of strain 3267 in the ELISPOT resulted in lower frequencies regardless of the strain used for challenge. In experiment 2, re-inoculation of PRRSV did not produce a clear pattern.

### Cytokine (IL-1, IL-6, IL-8, TNF-α, IL-10, and TGF-β) ELISAs

Cytokine levels in serum were examined only after the second challenge of each experiment and in naïve control pigs. Regarding IL-1, all samples were below the assay detection limit in both experiments. For IL-6, in experiment 1 two animals in each group (A + A and A + B) had detectable levels of this cytokine from day 1 to 7 post-challenge; naïve pigs inoculated for the first time produced negative results. In contrast, all samples yielded negative results for IL-6 in experiment 2.

For IL-8 (Table 7), differences were noticed among groups and treatments. In experiment 1, naïve pigs inoculated for the first time at day 84 with strain 3267 were positive for IL-8 (corrected concentration) at days 1, 2, 3 and 7 post-challenge. In contrast, in the HeC model IL-8 was only detected at days 1 and 2. In the HoC, at least one animal produced a positive result every examined day but no constant pattern was seen. In experiment 2, the picture was the opposite to that of experiment 1. Naïve animals inoculated for the very first time with 3262 produced IL-8 results below the assay detection limit at all points. In contrast, at least one animal had detectable IL-8 levels at any given time after the HoC or HeC. For TNF-α, no significant differences were noticed and for IL-10, in both experiments positive results were sporadic.

**Table 7:** Serum levels of IL-8 after challenge with PRRSV at 84 days after the initial inoculation

	**Corrected† IL-8 levels in serum (pg/mL)** **N° responding pigs; Average ± standard deviation**				
**Experiment 1** ** Group**	**Days post-challenge**				
	1*	2*	**3**	**7¶**	**14**
**C1 + B**	2/2^a^ 270 ± 108	2/2^a^ 424 ± 255	2/2^a^ 440 ± 434	2/2^a^ 470 ± 606	1/2 711 ± N.A.
**A + A**	0/4 N.A.	1/4 440 ± N.A.	2/4 272 ± 363	1/4 822 ± N.A.	1/4 124 ± N.A.
**A + B**	2/4 644 ± 479	3/4 194 ± 98	0/4 N.A.	0/4 N.A.	0/4 N.A.
**Experiment 2** ** Group**	**Days post-challenge**				
	**1**	**2**	**3**	**7**	**14¶**
**C2 + A**	0/3^b^ N.A.	0/3^b^ N.A.	0/3^b^ N.A.	0/3^b^ N.A.	0/3 N.A.
**B + B**	3/4 249 ± 192	1/4 526 ± N.A.	2/4 212 ± 211	2/4 262 ± 247	3/4 297 ± 273
**B + A**	2/3 38 ± 2	1/3 667 ± N.A.	2/3 1088 ± 407	1/3 577 ± N.A	3/3 291 ± 117

**†** In order to account the pre-2nd challenge levels of a given cytokine, values (pg/mL) obtained with samples of day 0 (84 pi) (C1 + B:653 ± 66; A + A:796 ± 306; A + B:1062 ± 157; C2 + A:503 ± 133; B + B:187 ± 198; B + A:41.3 ± 0) were subtracted to the values obtained at days 1, 2, 3, 7 or 14, respectively for producing a corrected value (Corrected value for cytokine X = Concentration of cytokine X _day n_ - Concentration of cytokine X _day 0_).

N.A. not applicable. Symbols (* *p* = 0.05; **¶***p* < 0.05) show days for which statistical differences were observed between groups of a given experiment with regards to the proportion of positive pigs. Letters indicate statistically significant differences for the proportion of responding pigs for similar groups when comparing between experiments; i.e. naïve pigs inoculated with 3267 (C1 + B) vs. naïve pigs inoculated with 3262 (C2 + A). In this case, a,b correspond to *p* ≤ 0.1.

For TGF-β (Table 8), a different outcome was seen depending on the experiment and group. Thus, in experiment 1, naïve pigs inoculated with 3267 were sporadically positive similarly to what occurred in naïve pigs inoculated with 3262 in experiment 2. In experiment 1, both in the HoC and in the HeC, re-inoculation with PRRSV induced increased levels of TGF-β that peaked at day 7 or 14 post challenge. In experiment 2, differences were seen depending on the strain used for re-inoculation. Thus, while in the HoC model all animals had sustained serum levels of TGF-β from day 1 to 14 post re-inoculation, in the HeC model TGF-β was detected only sporadically.

**Table 8:** Serum levels of TGF-β after challenge with PRRSV at 84 days after the initial inoculation

					
**Experiment 1** ** Group**	**Days post-challenge**				
	**1**	**2**	**3**	**7**	**14**
**C1 + B**	0/2	0/2	1/2	1/2	1/2
	N.A.	N.A.	74 ± N.A.	113 ± N.A.	23 ± 18
**A + A**	3/4	3/4	4/4	4/4	4/4
	50 ± 10	67 ± 17	46 ± 60	93 ± 14	152 ± 110
**A + B**	3/4	2/4	2/4	4/4^a^	4/4
	57 ± 60	115 ± 60	109 ± 50	99 ± 59	144 ± 90
**Experiment 2** ** Group**	**Days post-challenge**				
	**1**	**2**	3*	7*	**14**
**C2 + A**	1/3	1/3	0/3	1/3	1/3
	57 ± N.A.	149 ± N.A.	N.A.	17 ± N.A.	33 ± N.A.
**B + B**	4/4	4/4	4/4	4/4	4/4
	42 ± 29	42 ± 16	99 ± 41	66 ± 66	109 ± 140
**B + A**	2/3	2/3	0/3	0/3^b^	1/3
	106 ± 34	109 ± 61	N.A.	N.A	37 ± N.A.

**†** In order to account the pre-2nd challenge levels of a given cytokine, values (pg/mL) obtained with samples of day 0 (84 pi) (C1 + B:141 ± 20; A + A:58 ± 29; A + B:86 ± 63; C2 + A:124 ± 107; B + B:137 ± 14; B + A:1036 ± 1701) were subtracted to the values obtained at days 1, 2, 3, 7 or 14, respectively for producing a corrected value (Corrected value for cytokine X = Concentration of cytokine X _day n_ - Concentration of cytokine X _day 0_).

N.A. not applicable. Symbols (*p ≤ 0.05) show days for which statistical differences between groups with regards to the proportion of positive pigs were observed. Letters indicate statistically significant differences for the proportion of responding pigs for similar groups between experiments; i.e. naïve pigs inoculated with 3267 (C1 + B) vs. Naïve pigs inoculated with 3262 (C2 + A). In this case, a,b correspond to *p* ≤ 0.05.

## Discussion

The present study shows that two different PRRSV isolates of genotype I subtype I can produce two different models of infection based on the clinical, virological and immunological parameters examined. Although both models were absent of very overt clinical signs, in one of them (strain 3262) fever and the decrease in weekly weight gains were very mild compared to the other (strain 3267). Differences in the outcome of the inoculation attributable to the breed, genetics, to the origin of pigs or to different environmental conditions were controlled by using animals from a single farm with a common genetic and health background. The use of controlled environment BSL3 facilities reduced variability attributable to environmental conditions. Regarding the potential bias caused for concomitant secondary pathogens, the results make evident that some pigs in experiment 2 developed a bacterial infection, a fact that was not seen in experiment 1. This could have affected the clinical outcome of the infection after some days of clinical course but is difficult to elucidate the precise impact of secondary infections on the virological course or on virus-specific immune responses.

Regarding the virological outcome of the initial challenge, it was evident that strain 3267 produced longer and sustained viremias in piglets compared to what strain 3262 did. Although causes for those different outcomes cannot be precisely defined at present, those observations will be coherent with the higher replication rates of strain 3267 observed in vitro in PAM cultures compared to those of 3262 (10^7.0–7.5^ versus 10^5.0–5.9^ TCID^50^/mL after 48 h of incubation at similar m.o.i., data not shown). Also, persistence of the virus can have an individual component as evidenced by the fact that half of the pigs infected with strain 3267 resolved viremia by day 42 pi.

For the humoral response, as far as S/P values in ELISA are concerned, strain 3267 induced higher and more sustained S/P values than strain 3262. Although the nature of this fact is difficult to ascertain with the available data; it can be hypothesized that the higher sustained replication levels of 3267 could be the cause.

With regards to NA, in the present study it became evident that their role in clearing viremia during the course of the infection can be limited. In experiment 1 viremia ceased in absence of detectable NA against the homologous isolate and in experiment 2, viremia coexisted for weeks with NA. Glycosylation of neutralizing epitopes have been reported to result in a poorer development of NA or in increased difficulties for viral neutralization to occur [27]. Strain 3262 for which development of NA did not occur -or could not be demonstrated- had six potential glycosylation sites in GP3, the neutralization epitope (NE) in GP4 had a deletion and the assumed NE in GP5 contained three potential glycosylation sites (N-37, N-46 and N-53). Strain 3267 harbour six potential glycosylation sites in GP3, had an intact GP4 – very close to that of the reference Lelystad virus- and harbour two glycosylation sites in GP5 (N-46 and N-53) [15]. Additionally, when the virus 3267 present in blood of viremic pigs at day 49 pi was sequenced, a third glycosylation site in N-37 of GP5 was detected and GP4 had suffered some variations in comparison with the parental strain (data not shown). Thus differences in the levels of NA may arise from the different characteristics in the sequence and number of glycosylations of the NE of GP3 GP4 and GP5. On the other hand, when cross-neutralization experiments were performed, it was seen that sera of animals primo-inoculated with strain 3267 were also capable of neutralizing strain 3262 although with much lower efficiency. Sera raised against 3262 were devoid of neutralizing capabilities even against the homologous 3262. Considered globally, these results suggest that in PRRSV, under some circumstances heterologous NA could be more efficient than the homologous ones. This has been observed recently by Martínez-Lobo et al. [12]. The nature of this phenomenon is unknown. Classically, these differences would have been attributed to changes in the sequence of the NE and to the number of glycosylations present in those NE and actually, these changes occurred in the present study. Nevertheless, in the study by Martínez-Lobo et al. [12] differences in cross-neutralization in sera raised against different genotype I strains could not be related strictly to the sequence and number of glycosylations of the known GP3, GP4 or GP5 NE and those authors suggested that maybe the conformational characteristics of the epitopes could have a role on the cross-reactivity or, alternatively, that other NE unknown yet exist.

In any case, taking in consideration the abovementioned facts, if pre-formed NA play a role in protection against PRRSV re-infection as indicated by previous papers [5,9,27], animals infected/immunised initially by strain 3262 should be more likely infected upon the HoC or HeC than animals infected/immunized initially with 3267. This is what occurred and as shown in Tables 4 and 5: animals immunized with strain 3262 developed viremia after either the HoC or the HeC, although viremia was of low intensity.

When the cell-mediated compartment of the immune response was examined, it was evident that strain 3262 induced a stronger virus-specific response measured as IFN-γ-SC. Interestingly, this situation was not reversed by the second challenge at day 84 pi, even for animals primo-immunized with 3267 and challenged again with 3267. In this case the higher levels of replication of isolate 3267 did not seem to contribute to a higher cell-mediated response.

In a previous study [16] it was shown that infection of dendritic cells with strain 3267 resulted in an increase of SLA-II^+^/CD80/86^-^ and SLA-II^-^/CD8086^+^ cells while strain 3262 induced a decrease in the proportion of SLA-II^+^/CD80/86^+^ cells. Both 3262 and 3267 down regulated SLA-I in infected cells. Also, strains 3267 and 3262 induced different TNF-α and IL-10 responses in dendritic cells. These differences may lie behind the different immune responses developed after the first challenge.

Besides that, strain 3262 produced higher frequencies of IFN-γ SC when used either as homologous or heterologous recall stimulus in ELISPOT. This fact points to the potential differences in the respective capabilities of the examined strains to inhibit IFN-γ responses or to a different antigenicity of T-epitopes. Interestingly, after the second challenge, boost of IFN-γ SC responses were only seen for pigs showing frequencies lower than 100–150 cells per million both in experiment 1 and experiment 2, a fact that suggest that this value indicates saturation of the IFN-γ responses. To our knowledge this is the first report showing differences in cell-mediated responses against different PRRSV field strains.

When protection against a second challenge was examined, results showed that weight gains were always better in the HoC scenario. However, in virological terms, the picture was different. In experiment 1 (isolate 3262), in which pigs had low or non-detectable levels of neutralizing antibodies, the outcome of the HoC or HeC was similar regarding viremia but homologous protection was better when distribution of virus in tissues was considered (1/4 positive results in tonsil of pigs in the HoC versus 4/4 for the HeC).

Since NA against 3262 were not present, or were not detected, this would indicate that cell-mediated immunity most probably was responsible for limiting the spread of the infection and the duration of viremia. In experiment 2, where cell-mediated responses were lower but NA were higher, viremia was not detected by day 7 post second inoculation. However, that was not truly sterilizing immunity in all cases as some animals, both in the HoC and HeC models, harboured PRRSV in tonsils (but not lungs or spleens). In this second experiment protection was higher compared to experiment 1 but some animals did not withstand the challenge even in a homologous one. In a previous paper it was reported that protection in the homologous challenge model may occur with NA titres higher than 1:8 [9]; our results suggest that titres needed for complete protection probably have to be higher than considered before.

Overall, the results point towards the significance of each compartment of the immune response in protection against PRRSV and on the development of future vaccines. The results of the present study suggest that preformed NA may have a protective role against infection but animals devoid of detectable NA may limit the spread of the infection indicating thus that probably cell-mediated immunity also contributes to protection.

Regarding the systemic response of cytokines, results showed that clear differences only occurred for IL-8 and TGF-β. Although it is difficult to know, we hypothesize that the reason behind the lack of responses for the other examined cytokines might be related to the sample chosen (serum), to the sensitivity of the assay used, but also it may just reflect the reality. A review of published paper reveals that a considerable conflict exists measuring IL-10, IL-6 or TNF-α after a PRRSV infection. This controversy could be due to the different samples or to the different assay methods used to detect a given cytokine, but also to the viral isolate variability [3]. Similarity for IL-1, an up-regulation of this cytokine in broncho-alveolar lavages have been noticed after a PRRS infection [28,29], whereas other have observed an up-regulation of IL-1 in PBMC cultures but no in serum, as in our case, after the PRRSV infection [30,31]. Regarding IL-8, naïve animals challenged for first time with 3267 produced sustained levels of IL-8 for at least one week compared to the brief responses observed in re-inoculated pigs or in naïve pigs inoculated with 3262. Those sustained levels of IL-8 for 3267 strain administered to naïve pigs are suggestive of a more powerful inflammatory potential for that strain compared to 3262.

When TGF-β responses were examined in experiment 1, serum levels of this cytokine were observed mostly in immunized pigs challenged for the second time but only sporadically in naïve pigs inoculated anew, suggesting that TGF-β is produced in recall responses to PRRSV. In the case of pigs previously immunized with 3267, the HoC produced more sustained levels of serum TGF-β than the HeC perhaps a suggestion that epitopes involved in the generation of TGF-β producing T-cell clones can be at some extent dependent on the strain. This would provide an explanation for previous in vitro observations about the differential induction of regulatory T-cells by PRRSV [32].

With the present data, it is clear that almost opposite models of immune response to PRRSV could exist depending on the strain: one based mainly in the development of NA with low IFN-γ responses, the other with predominance of IFN-γ responses and a poor development of NA. Also, the study shows that in virological terms –but not based on zootechnical parameters- heterologous immunity sometimes could be more efficacious than the homologous one. This study contributes to emphasize the need for examining future PRRSV vaccines and PRRS immunopathogenesis studies considering the immunobiological diversity of PRRSV strains.

## Abbreviations

IFN-γ, Interferon gamma; IPMA, Immunoperoxidase monolayer assay; HeC, Heterologous challenge; HoC, Homologous challenge; NA, Neutralizing antibodies; NE, Neutralization epitope; pi, Post-inoculation; PAM, Porcine alveolar macrophages.

## Competing interests

The authors declare that they have no competing interests.

## Authors’ contributions

Conceived and designed the experiments: ID, MG, LD, MM, JP, EM. Performed the experiments: ID, MG, LD, NN, LK, SL, IG, JS. Analysed the data: ID, MG, EM, LD, MM. Contributed reagents/materials/analysis tools: ID, MG, NN, JP. Wrote the manuscript: ID, MG, MM, EM. Critical discussion and final approval of manuscript: ID, MG, LD, NN, LK, SL, IG, JS, MM, JP, EM. All authors read approved the final manuscript.
